# Reduction of transmission from malaria patients by artemisinin combination therapies: a pooled analysis of six randomized trials

**DOI:** 10.1186/1475-2875-7-125

**Published:** 2008-07-09

**Authors:** Lucy C Okell, Chris J Drakeley, Azra C Ghani, Teun Bousema, Colin J Sutherland

**Affiliations:** 1 Department of Infectious & Tropical Diseases, London School of Hygiene & Tropical Medicine, London, UK; 2 MRC Centre for Outbreak Analysis & Modelling, Department of Infectious Disease Epidemiology, Imperial College London, London, UK; 3 Department of Medical Microbiology, Radboud University Nijmegen Medical Centre, Nijmegen, The Netherlands

## Abstract

**Background:**

Artemisinin combination therapies (ACT), which are increasingly being introduced for treatment of *Plasmodium falciparum *malaria, are more effective against sexual stage parasites (gametocytes) than previous first-line antimalarials and therefore have the potential to reduce parasite transmission. The size of this effect is estimated in symptomatic *P. falciparum *infections.

**Methods:**

Data on 3,174 patients were pooled from six antimalarial trials conducted in The Gambia and Kenya. Multivariable regression was used to investigate the role of ACT versus non-artemisinin antimalarial treatment, treatment failure, presence of pre-treatment gametocytes and submicroscopic gametocytaemia on transmission to mosquitoes and the area under the curve (AUC) of gametocyte density during the 28 days of follow up.

**Results:**

ACT treatment was associated with a significant reduction in the probability of being gametocytaemic on the day of transmission experiments (OR 0.20 95% CI 0.16–0.26), transmission to mosquitoes by slide-positive gametocyte carriers (OR mosquito infection 0.49 95% CI 0.33–0.73) and AUC of gametocyte density (ratio of means 0.35 95% CI 0.31–0.41). Parasitological treatment failure did not account for the difference between ACT and non-artemisinin impact. The presence of slide-positive gametocytaemia prior to treatment significantly reduced ACT impact on gametocytaemia (p < 0.001). Taking account of submicroscopic gametocytaemia reduced estimates of ACT impact in a high transmission setting in Kenya, but not in a lower transmission setting in the Gambia.

**Conclusion:**

Treatment with ACT significantly reduces infectiousness of individual patients with uncomplicated falciparum malaria compared to previous first line treatments. Rapid treatment of cases before gametocytaemia is well developed may enhance the impact of ACT on transmission.

## Background

With resistance to chloroquine and sulphadoxine-pyrimethamine well-established in many malaria endemic countries, artemisinin combination therapies (ACT) are now being introduced for first line treatment of *Plasmodium falciparum *malaria [1,2]. Clinical trials of ACT demonstrate high efficacy [3] and no stable, naturally-occurring resistance has yet been reported. A potential further benefit is that the artemisinin derivative component of ACT has strong activity against gametocytes, the life stage of the parasite which is transmitted from humans to mosquitoes, at all but the most mature stages of their development [4-6]. This contrasts with previous first line treatments such as chloroquine and sulphadoxine-pyrimethmine, which had only limited activity against early developing gametocytes [7,8]. Furthermore ACT reduce the asexual parasite population, the source of new gametocytes, more swiftly [3]. By reducing the infectiousness of treated, symptomatic patients, ACT may be used to reduce overall transmission intensity [9-12] and the spread of parasite strains resistant to the drug combined with the artemisinin derivative [13,14]. To assess the potential for these population level effects, it is important to have an accurate estimate of the size of ACT impact on the infectivity of individual, symptomatic *P. falciparum *infections.

Previous summary analyses of randomized ACT trials have shown a reduction in gametocyte prevalence at single timepoints during follow up but have focussed on clinical efficacy [6,15]. Gametocyte density is important because it is correlated with transmission success to mosquitoes, although this relationship is variable [16]. The best measures of transmission are the proportion of mosquitoes infected by feeding on a patient blood sample [16-18], and the density of infection in those mosquitoes [16-18]. Here a pooled analysis of six trials is presented, comparing a range of ACT and non-artemisinin antimalarials with the focus on obtaining estimates of impact on gametocyte density over time and on infectivity of patients to mosquitoes. This includes to the best knowledge of the authors all published studies of mosquito infection by patients treated with ACT. The roles of treatment failure and pre-treatment gametocytaemia are investigated as these two factors are expected to influence the size of ACT impact on transmission as they are rolled out in malaria-endemic regions.

## Methods

### Data

Raw data were pooled from six previously published randomized antimalarial trials in which participants were assessed for infectivity to mosquitoes after treatment (Table 1) [13,19-22]. Five of the trials were carried out in the Gambia during the four month season of intense transmission (September – December, entomological inoculation rate (EIR) ~3–50 infectious bites per person per year (ibppy) [23]) over five successive years (1998–2002) and one in Kenya in an area of high, perennial transmission during 2003–2004 (EIR ~70 ibppy). The trials were approved by the relevant ethics committees: the Joint Gambia Government/Medical Research Council [13,20-22], the London School of Tropical Medicine and Hygiene [13,20,21], and the Kenya Medical Research Institute/National Ethical Review Committee [19], and complied with the revised Helsinki Declaration of 1983.

**Table 1:** Baseline characteristics by treatment group and study at day 0. Means are arithmetic. CQ = chloroquine, SP = sulphadoxine-pyrimethamine, AS1 = 1-dose artesunate, AS-3 = 3-dose artesunate, AL = artemether-lumefantrine (Coartem), AQ = amodiaquine.

Study & country	Treatment	N	Parasitology follow up days (bold = transmission experiment)	Mean age (years)	Mean asexual parasite density/μl	Mean slide- positive gametocyte prevalence (%)	Mean gametocyte density/μl
1. Gambia Targett 2001 [22]	CQ	135	**4**, **7**,14,28	5.9	80,886	2.5	33
	SP	277		6.0	73,742	5.6	13
	SP-AS1	113		6.2	23,147	10.7	10
	SP-AS3	74		6.3	26,567	13.4	88
	*all*	*599*		*6.0*	*59,581*	*6.9*	*26*
2. Gambia Targett 2001 [22]	SP	101	**7**,14	4.4	28,684	6.0	31
	SP-AS3	404		4.8	27,738	8.0	127
	*all*	*505*		*4.7*	*27,927*	*7.6*	*107*
3. Gambia Drakeley 2004 [20]	CQ	129	3, **7**,14,28	4.7	64,329	20.2	10
	CQ-AS	386		4.8	66,593	20.2	22
	*all*	*515*		*4.8*	*66,026*	*20.2*	*19*
4. Gambia Hallett 2006 [13]	CQ	125	3, **7**, **10**, **14**,28	4.5	63,640	21.6	30
	SP	180		4.5	58,128	23.2	47
	CQ-SP	193		4.6	79,900	19.2	37
	*all*	*498*		*4.6*	*68,009*	*21.2*	*39*
5. Gambia Sutherland 2005 [21]	CQ-SP	89	**7**,14,28	4.3	56,444	4.5	5
	AL	400		4.4	57,550	6.3	9
	(Coartem)						
	*all*	*489*		*4.3*	*57,347*	*6.0*	*9*
6. Kenya Bousema 2006 [19]	SP	152	3,7, **14**,28	3.4	19,044	20.9	7
	SP-AQ	127		2.9	20,578	22.3	8
	SP-AS3	174		3.2	21,620	24.4	8
	AL (Coartem)	75		4.0	21,178	24.6	6
	*all*	*528*		*3.3*	*20,565*	*22.9*	*7*

The study design and laboratory methods for each trial are described in detail elsewhere [19-22,24]. In brief, in each trial patients with uncomplicated microscopy-confirmed *P. falciparum *malaria with >500 asexual parasites/μl who sought treatment at a health centre were randomized to treatment groups. Altogether four non-artemisinin antimalarial regimens were tested: chloroquine (CQ), sulphadoxine-pyrimethamine (SP), CQ-SP, sulphadoxine-pyrimethamine-amodiaquine (SP-AQ) and four ACT: chloroquine-artesunate (CQ-AS), SP-AS1 (single dose of artesunate), SP-AS3 (3 days artesunate) and artemether-lumefantrine (AL). Dosage schedules were as follows: study 1: CQ: 10 mg/kg days 0–3, SP: 250 mg sulphadoxine +12.5 mg pyrimethamine if ≤ 10 kg, plus 125 mg sulphadoxine + 6.25 mg pyrimethamine per 5 kg increase in bodyweight day 0, SP-AS1: as SP, plus 4 mg/kg AS day 0, SP-AS3: as SP, plus 4 mg/kg AS days 0–2; study 2: SP & SP-AS3: as study 1; study 3: CQ: 10 mg/kg days 0–1, 5 mg/kg day 2, CQ-AS: as CQ, plus 4 mg/kg AS days 0–2; study 4: CQ: as study 3, SP: 25 mg/kg sulphadoxine, 1.25 mg/kg pyrimethamine day 0, CQ-SP: as CQ & SP; study 5: CQ-SP: CQ as study 1 days 0–2 plus SP day 0 as study 1, AL: 20 mg artemether plus 120 mg lumefantrine per 5 kg body weight up to 24 kg, or 120 mg artemether plus 720 mg lumefantrine for 25 kg+ 0 h, 8 h, 20 h, 32 h, 44 h, 56 h; study 6: SP: as study 4, SP-AQ: as SP plus 10 mg/kg AQ days 0–2, SP-AS3: as SP plus 4 mg/kg AS days 0–2, AL: as study 5.

Giemsa-stained blood sample smears were made on a number of days during follow-up until day 14 or day 28 after treatment (Table 1). Presence and density of asexual parasites and gametocytes were assessed separately in 100 high-powered microscopy fields assuming 1 parasite per high-powered field is equivalent to 500 parasites/μl (studies 1–5), or against white blood cells (WBCs), assuming 8000 WBCs/μl (study 6). In study 6, a more sensitive molecular technique (RNA-based real-time nucleic acid sequence-based amplication technique (QT-NASBA) [25]) was used in addition to microscopy. Parasitological treatment failure was defined as any slide positive for asexual parasites from day 7 onwards, or an asexual parasite density on day 3 or 4 which was 10% or more of the density on day 0. Individuals in study 2, where the follow up was only 14 days, and those with any missing follow up parasitology data were excluded from analyses involving treatment failure. In addition, clinical failure data was available for 3 studies (3, 5 & 6), defined as patients who had parasitaemia and a fever (axillary temperature ≥ 37°C) at any time during follow up.

Venous blood samples for human-to-mosquito transmission experiments were taken on a specified day of follow-up (either 4, 7, 10 or 14, Table 1) from patients who were slide-positive for gametocytes by Field's stain (all studies). An additional group of randomly-selected patients donated blood for transmission experiments regardless of gametocyte carriage in studies 5 & 6 only. Membrane-feeding of *Anopheles gambiae *was performed as previously described [19-22,24]. In brief, blood samples were centrifuged and the red blood cell pellet was resuspended in the patient's own (autologous) plasma and separately in control serum from malaria-naïve volunteers in studies 1–5. In study 6 blood samples were used without further preparation. Female *Anopheles gambiae*, who were either the F1 progeny of wild-caught gravid females (Studies 1–4), or from a laboratory colony (studies 5 & 6), were fed on blood samples through an artificial membrane at 37°C. Mosquito midguts were dissected at 7–8 days and the number of oocysts in the mosquito midgut was determined by microscopy.

### Statistical methods

Four outcomes were considered: 1) the area under the gametocyte density-time curve (AUC), 2) the proportion of patients with slide-positive gametocytaemia on the day of mosquito feeding, 3) the proportion of mosquitoes infected with oocysts (the most readily quantifiable parasite stage in the mosquito), and 4) oocyst density in each dissected mosquito midgut. Asexual parasite and gametocyte densities are reported as arithmetic rather than geometric means to take into account zero values and the variability of the data. The AUC for each individual was calculated by taking the arithmetic mean of adjacent timepoints and multiplying by the intervening number of days, using data from days 0, 7, 14 and 28. Individuals missing gametocyte density readings on day 0, day 28, or at more than one other timepoint were not included in the AUC analysis.

Individual treatments were grouped into ACT and non-artemisinin antimalarials. One treatment regimen which contained only 1 dose of an artemisinin derivative (SP-AS1, study 1) was grouped separately. Antimalarials which were used in at least two studies were also examined individually; for the remainder, treatment effect could not be well distinguished from study effect. Gametocyte prevalence at day 0 and parasitological treatment failure were investigated as modifiers of ACT effect on all four outcomes.

The impact of ACT and other covariates on the AUC (outcome 1) was analyzed using negative binomial regression, which models the log of the arithmetic mean and gives a good fit to the skewed distribution [26]. Regression coefficients can be interpreted as ratios of arithmetic means. A term was included for random study effects. Densities of zero were included. The proportion of patients with slide-positive gametocytaemia on the day of mosquito feeding (outcome 2) was analysed by logistic regression of patient level data with random study effects. Finally, multilevel logistic regression at the mosquito level incorporating patient and study random effects was used to analyse the effect of ACT and other covariates on the presence of infection in the mosquito (outcome 3) and the presence of a high density oocyst infection, defined as greater than the median of positive oocyst counts (>4, outcome 4). Mosquito-level outcomes could not be adjusted for baseline factors because the majority of transmission experiments included only patients who were gametocyte positive at the time of feeding. Any baseline factors influencing transmission to mosquitoes also influence gametocytaemia and so inclusion into the dataset. The analysis was therefore adjusted only for the type of serum used (autologous versus control).

All analyses were undertaken using Stata (version 9.2, StataCorp LP).

## Results

**Figure 1:**
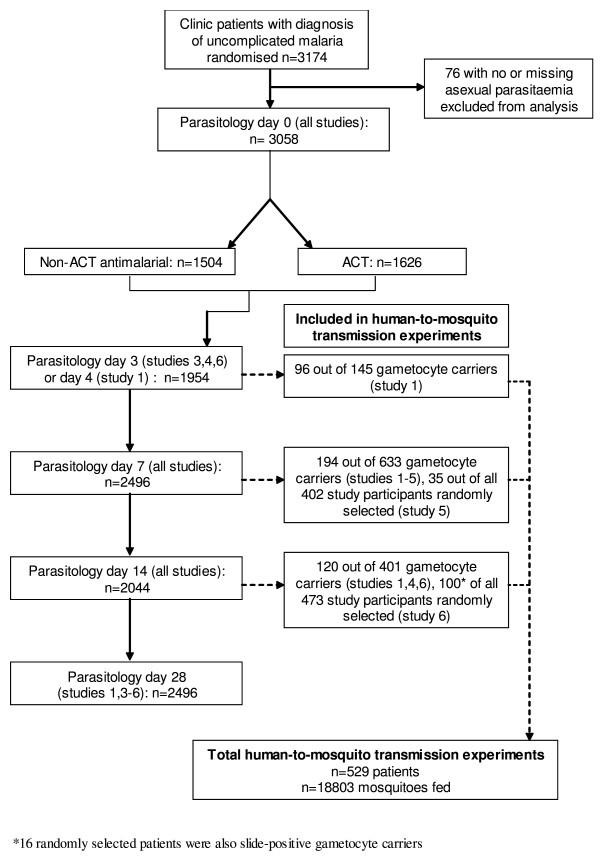
Overview of trial process and pooled data from the six studies.

Data were available on 3,174 patients in the six trials, of whom 529 contributed data to the human-to-mosquito transmission results (Figure 1). Baseline age, asexual parasitaemia, gametocytaemia and numbers of mosquitoes fed per person were significantly different between the studies (p < 0.05), but treatment groups within studies were comparable (Table 1). Eighty six participants, (2.7%) were estimated to have >500 asexual parasites/μl on the initial Field's-stained slide but were subsequently found to fail this inclusion criterion after examination of the confirmatory Giemsa-stained slide. 111 (27.1%) gametocyte donors for transmission experiments similarly fit the criterion of gametocyte slide-positivity by the Field's-stained slide only. Those with zero or missing asexual parasitaemia on day 0 were excluded (n = 76), but the remainder were retained in the analysis.

### Gametocytaemia during follow-up and transmission to mosquitoes

Outcomes are shown by study and treatment group in Table 2 and the pooled analysis is shown in Table 3.

**Table 2:** Gametocytaemia and mosquito level outcomes by treatment group and study.

Study & country	Treatment	Parasitological treatment failure (%)	Mean AUC per day	% gametocyte slide-positive at transmission experiment	N transmission experiments (patients)	% mosquitoes infected*	Mean oocyst density
1. Gambia Targett 2001 [22]	CQ	59.3	67.4	17.4	21	13.4	3.1
	SP	12.9	117.6	37.8	57	5.5	0.5
	SP-AS1	3.8	3.4	16.3	22	8.7	0.5
	SP-AS3	0	11.9	25.4	12	18.8	5.2
	*all*	*23.9*	*66.2*	*27.6*	*112*	*9.0*	*1.6*
2. Gambia Targett 2001 [22]	SP	-†	-†	64.0	20	10.7	1.5
	SP-AS3			13.4	20	6.1	0.3
	*all*			*23.3*	*40*	*8.4*	*0.9*
3. Gambia Drakeley 2004 [20]	CQ	73.5	58.7	49.5	35	13.1	3.9
	CQ-AS	54.7	36.4	26.5	32	5.2	0.4
	*all*	*59.6*	*42.0*	*32.1*	*67*	*9.3*	*2.1*
4. Gambia Hallett 2006 [13]	CQ	73.5	81.1	54.2	17	5.5	0.8
	SP	57.4	216.2	89.6	30	2.7	0.2
	CQ-SP	19.4	134.8	61.3	20	4.6	1.1
	*all*	*46.0*	*147.7*	*64.8*	*67*	*4.0*	*0.6*
5. Gambia Sutherland 2005 [21]	CQ-SP	19.0	44.6	41.7	22	3.0	0.1
	AL	15.0	2.9	5.8	39	0.1	0.0
	(Coartem)						
	*all*	*15.7*	*9.9*	*12.2*	*61*	*1.2*	*0.1*
6. Kenya Bousema 2006 [19]	SP	54.1	19.2	37.2	71	7.0	-‡
	SP-AQ	13.4	11.2	31.6	41	4.7	
	SP-AS3	17.9	3.9	9.6	41	2.6	
	AL	4.1	2.3	6.8	29	4.1	
	(Coartem)						
	*all*	*25*	*9.1*	*21.7*	*182*	*5.0*	

Means are arithmetic. CQ = chloroquine, SP = sulphadoxine-pyrimethamine, AS1 = 1-dose artesunate, AS-3 = 3-dose artesunate, AL = artemether-lumefantrine (Coartem), AQ = amodiaquine. AUC = area under the gametocyte density-time curve. For numbers of patients contributing to parasitology outcomes see Table 1.

* mean of percentages per patient

† follow up was only 14 days compared to 28 days in the other studies. Therefore study 2 was not included in analyses involving parasitological failure or the AUC

‡ Oocyst count data was not collected

**Table 3:** Treatment effect on four transmission outcomes:

**Patient-level outcomes (gametocytaemia)**						
Variable	N patients	Univariate AUC Ratio of arithmetic means (95% CI)	P value	N patients	Univariate OR slide- positive gametocytaemia on day of feeding (95% CI)	P value

**Antimalarial**						
CQ (non-artemisinin)	240	1	<0.00	337	1	<0.00
SP (non-artemisinin)	325	1.56 (1.27–1.93)	1	520	3.11 (2.19–4.40)	1
CQ-SP (non-artemisinin)	198	1.03 (0.81–1.32)		227	1.33 (0.86–2.06)	
CQ-AS (ACT)	291	0.72 (0.57–0.91)		344	0.42 (0.28–0.62)	
SP-AS3 (ACT)	187	0.62 (0.47–0.83)		581	0.50 (0.32–0.78)	
AL (Coartem) (ACT)	407	0.18 (0.13–0.25)		404	0.15 (0.08–0.29)	

**ACT**						
non-artemisinin	872	1	<0.00	1201	1	<0.00
ACT	885	0.35 (0.31–0.41)	1	1329	0.20 (0.16–0.26)	1

**ACT by pre-treatment gametocytaemia**						
*day 0 slide-negative*		†			†	
non-artemisinin	727	1	<0.00	994	1	<0.00
ACT	745	0.23 (0.19–0.28)	1	1125	0.13 (0.10–0.17)	1
*day 0 slide-positive*						
non-artemisinin	145	1	0.054	164	1	
ACT	140	0.80 (0.64–1.00)		183	0.41 (0.25–0.67)	<0.00

**ACT by parasitological treatment failure**						
*No failure*		†				
non-artemisinin	459	1	<0.00	468	1	<0.00
ACT	605	0.31 (0.25–0.37)		615	0.28 (0.20–0.38)	1
*Failure*					1	
non-artemisinin	269	1	<0.00	303		<0.00
ACT	218	0.51 (0.40–0.65)	1	233	0.33 (0.22–0.49)	1

**Mosquito-level outcomes**						

Variable	N mosquito es	Adjusted OR mosquito infection (95% CI)*	P value	N mosquito es	Adjusted OR oocyst count per midgut in highest 50% of positive counts (4) (95% CI)*	P value

**Antimalarial**						
CQ (non-artemisinin)	2194	1	0.002	2194	1	<0.00
SP (non-artemisinin)	6762	0.42 (0.20–0.87)		4079	0.25 (0.09–0.75)	1
CQ-SP (non-artemisinin)	1277	1.28 (0.43–3.85)		1146	0.12 (0.03–0.58)	
CQ-AS (ACT)	974	0.28 (0.11–0.71)		974	0.25 (0.05–1.21)	
SP-AS3 (ACT)	2793	0.29 (0.12–0.69)		1439	0.39 (0.09–1.8)	
AL (Coartem) (ACT)	2299	0.22 (0.08–0.61)		292	*- *‡	

**ACT**						
non-artemisinin	11653	1	0.029	7419	1	0.165
ACT	6066	0.49 (0.33–0.73)		2705	0.45 (0.14–1.39)	

**ACT by pre-treatment gametocytaemia**						
*day 0 slide-negative*						
non-artemisinin	8811	1	0.002	5700	1	0.008
ACT	4039	0.49 (0.31–0.77)		1419	0.11 (0.02–0.56)	
*day 0 slide-positive*						
non-artemisinin	2277	1	<0.00	1326	1	0.102
ACT	1870	0.30 (0.16–0.55)	1	1129	0.28 (0.06–1.29)	

**ACT by parasitological treatment failure**						
*No failure*						
non-artemisinin	4773	1	0.003	2037	1	0.486
ACT	3496	0.46 (0.28–0.76)		740	0.49 (0.07–3.62)	
*Failure*						
non-artemisinin	3027	1	0.021	1574	1	0.294
ACT	897	0.37 (0.16–0.86)		572	0.33 (0.04–2.59)	

at the patient level, the area under the curve of gametocyte density (microscopy measurement) during follow up (28 days), the presence of slide-positive gametocytaemia on the day of feeding; at the mosquito level, the presence of infection in mosquitoes, and high density infection in mosquitoes.

* adjusted for type of serum (autologous/control) used in transmission experiment

† P interaction with ACT/non-artemisinin <0.05

‡ Mosquitoes were infected only in study 6 where oocyst data was not collected, except one mosquito in study 5 which had a missing oocyst count.

#### Antimalarial treatment

**Figure 2:**
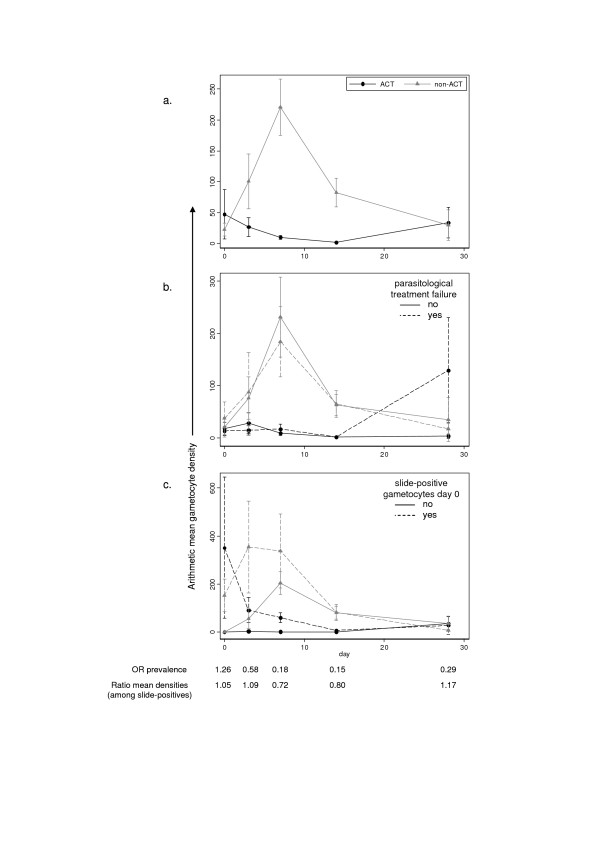
**Mean gametocyte density/μl during follow up in ACT (black, circles) and non-artemisinin (grey, triangles) antimalarial treatment groups, pooled results from the six trials (a) overall (b) stratified by parasitological treatment failure (c) stratified by presence of pre-treatment gametocytes.** Error bars show 95% confidence intervals. Lines drawn in between data points are theoretical only. Below: ratios showing ACT impact on gametocyte prevalence and density on each follow up day, allowing for random study effects.

Compared to non-artemisinin antimalarials, ACT significantly reduced the gametocyte AUC during follow up (ratio of means 0.35 95% CI 0.31–0.41, Figure 2a), gametocyte prevalence on day of feeding (OR 0.20 95% CI 0.16–0.26), and transmission to mosquitoes by slide-positive gametocyte carriers (OR mosquito infection 0.49 95% CI 0.33–0.73). The prevalence of high density oocyst infections was also reduced although this was not statistically significant (Table 3). Adjusting the first two outcomes for baseline asexual parasite density, gametocyte density, age group, month of transmission season and parasitological treatment failure did not substantially change the estimates (ratio of AUC means 0.38 95% CI 0.32–0.47, gametocytes on feed day OR 0.15 95% CI 0.12–0.19) (results of human-to-mosquito transmission experiments were not adjusted for these factors – see methods). The lower gametocytaemia in ACT-treated patients was consistent across studies whereas there was some variability in the proportions of mosquitoes infected and oocyst densities (Table 2). These outcomes were heavily skewed and strongly influenced by small numbers of highly infectious individuals in each treatment group.

The reduction in arithmetic mean gametocyte density in the ACT group compared to the non-artemisinin group varied over time during follow-up (Figure 2), being most marked up to day 7 and returning to non-significance by day 28. The same pattern was observed when the analysis was restricted to slide-positive gametocyte carriers at each time point i.e. those who could have been selected if a transmission experiment had been carried out then.

Individual treatment regimens varied in their impact on transmission outcomes. Compared to CQ, individuals treated with SP had significantly higher gametocytaemia during follow up, but slide-positive gametocyte carriers were less infectious to mosquitoes (Table 3). CQ-SP had a similar impact to CQ but was more effective at reducing high oocyst densities. All ACT treatment groups showed reduced gametocytaemia and lower prevalence of infection among mosquitoes compared to any non-artemisinin group. This was not seen with respect to oocyst densities. Within the individual trials however, only the SP-AS3 group in study 1 did not show reduced oocyst density (Table 2) compared to the non-artemisinins tested in the same trial. The artemether-lumefantrine (AL) regimen which contained 6 doses of artemisinin derivative had a significantly greater impact on gametocytaemia compared to the 3-dose regimens CQ-AS and SP-AS (prevalence at day of feeding: p = 0.001 and overall AUC: p < 0.001, respectively) and was associated with lower transmission to mosquitoes although this was not statistically significant (p = 0.460) (Table 3). Detectable asexual parasitaemia was also cleared more quickly in ACT-treated patients: 3% had positive slides on day 3 compared to 22% among those treated with non-artemisinin (p < 0.001).

#### Treatment failure

**Figure 3:**
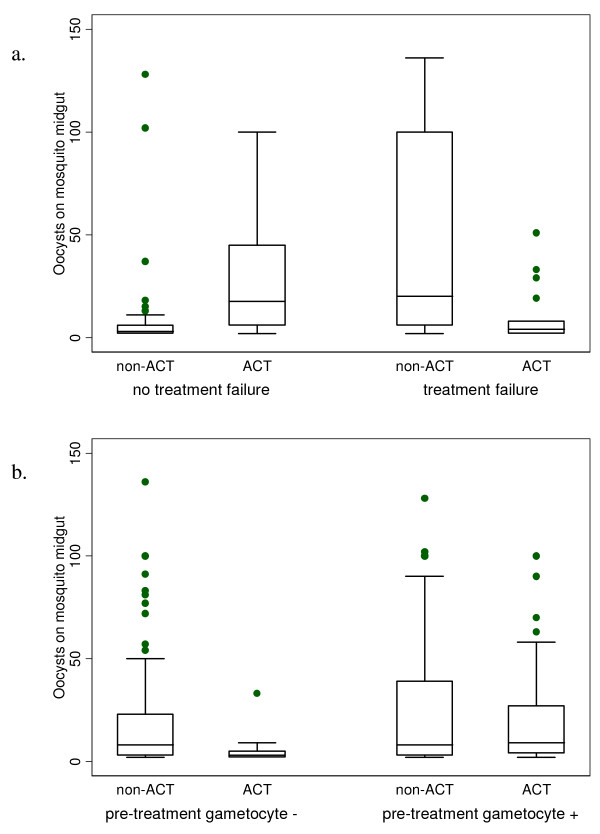
Box plots showing oocyst densities by ACT and (a) parasitological treatment failure and (b) pre-treatment gametocytes.

Parasitological failure by day 28 was more common among those receiving non-artemisinin treatment (42%) compared to ACT-treated individuals (28%, Table 2). However, this did not account for any of the impact of ACT on transmission outcomes, which remained very similar when parasitological failures were excluded: ratio of gametocyte AUC means = 0.31 (95% CI 0.25–0.37) (Figure 2b), OR gametocyte prevalence on the day of feeding = 0.28 (95% CI 0.20–0.38), OR of mosquito infection by slide-positive gametocyte carriers *= *0.46 (95% CI 0.28–0.76) and OR of high oocyst density 0.49 (0.07–3.62) (Figure 3a). This may be because parasitological failure often occurred late in follow-up; 21% of failures were detected at day 14, and 51% at day 28. These individuals may have been more infectious at later timepoints than those contributing to transmission experiments. In the studies where clinical failure was measured (3, 5 & 6), only 26% of parasitological failures (91/354) developed clinical symptoms (42% in the non-artemisinin group and 16% in the ACT group).

#### Pre-treatment gametocytes

Slide-positive gametocytaemia on day 0, indicating the presence of mature gametocytes before treatment, was predictive of higher gametocytaemia during follow up. ACT impact on gametocytaemia was reduced among those with pre-treatment gametocytes (ratio of gametocyte AUC means 0.80 (95% CI 0.64–1.00) and OR of gametocyte prevalence on the day of feeding 0.41 (95% CI 0.25–0.67) compared to those negative for pre-treatment gametocytes (ratio of gametocyte AUC means 0.23 (95% CI 0.19–0.28) and OR of gametocyte prevalence on the day of feeding 0.13 (95% CI 0.10–0.17) (interaction p < 0.001 for both outcomes) (Figure 2c). The pattern was similar for the oocyst density outcome, although the difference between the effects of the ACT and non-artemisinin groups was not significant (Figure 3b, Table 3). ACT impact on transmission to mosquitoes by slide-positive gametocyte carriers was similar among those with and without pre-treatment gametocytes (Table 3).

#### Submicroscopic gametocytaemia

In study 6, the molecular QT-NASBA technique detected gametocytes in 311/332 (94%) of blood samples that were classified gametocyte-positive by microscopy, and furthermore in 615/959 (64%) of those classified negative, giving a higher estimated prevalence of gametocytes at all timepoints [19]. Gametocyte density readings matched poorly between the two techniques so prevalence data is used to compare them.

Among patients who were tested by both QT-NASBA and microscopy (n = 249), ACT impact on gametocytaemia was smaller if submicroscopic gametocyte carriers were taken into account. The ratio of means of the area under the gametocyte prevalence-time curve comparing ACT to non-artemisinin antimalarials was 0.40 (95% CI 0.23–0.69 p < 0.001) using microscopy data, compared to 0.60 (95% CI 0.51–0.70 p < 0.001) with QT-NASBA data. This difference suggests bias is introduced by microscopy because a greater proportion of ACT-treated gametocyte carriers have a density in the submicroscopic range.

In study 5 in The Gambia, 1/1165 (0.1%) mosquitoes were infected when feeds were performed on 33 individuals who were gametocyte slide-negative, compared to 19/882 (2.2%) mosquitoes who were infected by 26 gametocyte carriers in the same study. This suggests that infection of mosquitoes by submicroscopic gametocytaemia was rare in this setting. By contrast, gametocyte slide-negative individuals in study 6 in Kenya infected almost as high a proportion of mosquitoes as gametocyte slide-positive patients: 4.7% compared to 5.9%. In this study the impact of ACT was substantially smaller if "being infectious" was defined as infecting any mosquitoes (regardless of microscopic gametocytaemia), rather than as being gametocyte slide-positive on the day of feeding (OR infecting any mosquitoes at feeding 0.42 95% CI 0.18–0.97 versus OR gametocyte prevalence at feeding = 0.14 95% CI 0.04–0.44).

## Discussion

These results demonstrate that gametocytaemia among ACT-treated patients is significantly lower than in individuals treated with non-artemisinin antimalarials during 28 days of follow up. ACT treatment is also associated with reduced human-to-mosquito transmission both among those with microscopically detectable gametocytes, and randomly selected patients. ACT appear to destroy a substantial proportion of immature, developing gametocytes while sequestered in the microvasculature, resulting in a significant reduction in the release of mature gametocytes into the peripheral blood. Their more rapid reduction of the asexual reservoir may also contribute.

Transmission to mosquitoes was measured mainly at day 7 when ACT impact on gametocytaemia was at one of its highest points. Although gametocyte density is not the only factor determining transmission to mosquitoes (maturity and sex ratio of gametocytes are also important [17,27]), the AUC analysis may give an estimate more representative of the overall impact during the follow up period. Longitudinal feeding studies would also be valuable as the infectiousness of gametocytes may change over the course of an infection [24].

Higher levels of parasitological treatment failure among non-artemisinin treated individuals did not account for any of the higher observed ACT impact on gametocytaemia and transmission to mosquitoes. However, parasitological failure did predict higher gametocytaemia and transmission to mosquitoes. Drug resistant parasite genotypes have been shown to enhance transmission by boosting gametocyte numbers, particularly in patients treated with monotherapies [13,20]. The late appearance of asexual parasites among treatment failures would mean that most of the resulting gametocytes would become patent and transmit after the 28 day follow-up period. Low rates of clinical symptoms among parasitological failures, at least during the period of follow up, suggests these individuals may not receive further treatment and so would subsequently be important contributors to transmission. ACT impact was slightly higher among treatment failures than successes, which may reflect a higher proportion of new parasite inocula as opposed to recrudescent infections in the ACT treatment failure group, which had not yet produced gametocytes [28]. ACT were significantly less able to reduce gametocytaemia among individuals who were slide-positive for gametocytes on presentation, probably because they do not act against mature gametocytes [5]. The pattern of gametocyte density over time among these patients was consistent with that of natural decay of mature gametocytes and limited subsequent release of developing gametocytes from sequestration. Duration of symptoms prior to treatment seeking has been shown to have a strong relationship with gametocyte prevalence at baseline [29]. Interventions to improve the speed of treatment seeking could therefore enhance the impact of ACT on transmission. However, older asymptomatic infections also contribute to the presence of gametocytaemia at baseline [29] and therefore ACT impact may be reduced in high transmission settings or towards the end of a period of seasonal transmission.

Study 6 in Kenya [19] highlighted the importance of submicroscopic gametocytaemia in studies of human infectiousness. Taking this into account reduced the estimate of ACT impact on gametocytaemia and in particular transmission to mosquitoes. However, study 5 in the Gambia showed little evidence of transmission from submicroscopic carriers [21]. The reason for this difference in findings is not clear. Differences in laboratory procedures are unlikely to be accountable because the infectiousness of gametocyte slide-positive individuals is comparable between the studies. The lower intensity of transmission in the Gambia, and concomitant seasonal fluctuations in gametocyte carriage are probably important [30]. However, high levels of submicroscopic gametocytaemia have been found in other low transmission settings, although the infectiousness of these individuals was not tested [31]. Further research is needed to determine the relationship between submicroscopic gametocytaemia, infectivity, and transmission setting.

The grouping of different treatments into non-artemisinin and ACT categories in these results is simplistic since individual treatment regimens within these groups sometimes had significantly different associations with the outcomes (Table 3). Therefore our estimates of ACT effect depend to some extent on the numbers of patients treated with each regimen in these studies. Some evidence exists that non-artemisinin antimalarials stimulate the post-treatment release of immature gametocytes to different extents [8,32], although these may not be sufficiently mature to infect mosquitoes [24], whilst other evidence suggests the gametocytaemia is no different from what would be expected in an untreated infection [33,34].

While it is useful to have a pooled estimate of ACT impact from these studies, the heterogeneity in results should not be overlooked. The laboratory methods used in each of the studies were mostly very similar, but there were some variations which may have influenced the estimated ACT impact, such as the number of days between treatment and measurement of human-to-mosquito transmission, and different microscopists. For example, the SP-AS3 treatment group in study 1 did not show reduced oocyst density among blood-fed mosquitoes compared to non-artemisinin treatments, but only 12 patients in this group participated in feeding experiments. Of the 6 individuals successfully infecting mosquitoes at day 4, two were already harbouring gametocytes at day 0, and a third had very high day 4 gametocytaemia. These individuals almost certainly carried gametocytes on day 4 representative of a substantial pre-treatment gametocyte population, and may have been less infectious if transmission experiments were performed 7 or 14 days after treatment, as in the other studies analysed. Differences in baseline characteristics between studies such as transmission setting could also have caused differences in ACT effects.

These results are representative of ACT effects upon young symptomatic patients in a trial setting. In order to evaluate potential impact of ACT on population-level transmission, it will be crucial to know the proportion of infections which are asymptomatic and so untreated in different transmission settings, and their relative contribution to the infectious reservoir. Many other operational factors including the coverage of ACT relative to other antimalarials and levels of adherence to treatment regimens will also be important in determining such effects. Empirical observations on population gametocyte carriage and its evolutionary basis [18,35] will aid this more comprehensive evaluation. Ongoing monitoring of the impact of ACT on transmission could be an important surveillance tool, as the early signs of spreading resistance to these regimens are likely to be enhanced transmission from treated individuals [24].

## Conclusion

ACT reduce gametocytaemia and onward transmission to mosquitoes significantly compared to previous first-line non-artemisinin antimalarials, although transmission from ACT-treated patients is not fully prevented. Malaria transmission intensity could potentially be reduced as ACT are scaled up in malaria-endemic countries, however it will be important to quantify the proportion of asymptomatic and therefore untreated infections in different populations. ACT impact is likely to be reduced by factors which increase the presence of mature gametocytes before treatment, such as lack of rapid access to treatment or high malaria transmission intensity. Future studies of different antimalarials should also measure the infectiousness of gametocyte carriers with submicroscopic densities, to further clarify the contribution of such individuals to post-treatment transmission.

## Authors' contributions

LCO designed the analysis with ACG, carried it out, and drafted the manuscript, ACG also revised the manuscript, CJD and CJS conceived the idea of a summary analysis, CJD, CJS and TB provided data from the original trials, revised the manuscript critically and aided the interpretation.
